# Dentists’ Working Conditions during the First COVID-19 Pandemic Lockdown: An Online Survey

**DOI:** 10.3390/healthcare9030364

**Published:** 2021-03-23

**Authors:** Vera Wiesmüller, Emanuel Bruckmoser, Ines Kapferer-Seebacher, Katharina Fink, Sabrina Neururer, Dagmar Schnabl, Johannes Laimer

**Affiliations:** 1 University Hospital for Operative and Restorative Dentistry, Medical University of Innsbruck, Anichstr. 35, 6020 Innsbruck, Austria; vera.wiesmueller@i-med.ac.at (V.W.); ines.kapferer@i-med.ac.at (I.K.-S.); katharina.fink@i-med.ac.at (K.F.); dagmar.schnabl@tirol-kliniken.at (D.S.); 2 Private Practice for Oral and Maxillofacial Surgery, 5020 Salzburg, Austria; research@bruckmoser.info; 3 Department of Medical Statistics, Informatics and Health Economics, Medical University of Innsbruck, Schoepfstr. 41/1, 6020 Innsbruck, Austria; S.Neururer@tirol-kliniken.at; 4 Department of Clinical Epidemiology, Tyrolean Federal Institute for Integrated Care, Tirol Kliniken GmbH, 6020 Innsbruck, Austria; 5 University Hospital for Craniomaxillofacial and Oral Surgery, Medical University of Innsbruck, Anichstr. 35, 6020 Innsbruck, Austria

**Keywords:** SARS-CoV-2, COVID-19, lockdown, online survey, questionnaire, dentistry

## Abstract

The aim of this study was to investigate the operating conditions of dentists in Central Europe during the first coronavirus disease 2019 (COVID-19) lockdown. A survey including 24 questions was emailed to dentists in Austria, Germany, Switzerland and South Tyrol (Italy). Questions regarding dentists’ field of work, working hours, treatments performed, personal protective equipment and protocols, and economic consequences were asked. 1731 participants were included. 30.4% of participants worked mainly in Austria, 60.8% in Germany, 6% in Switzerland and 2.1% in South Tyrol. A country-specific analysis for the situation of South Tyrol was not possible due to the low participation; 53.7% of German, 45.5% of Austrian, and 11.7% of Swiss respondents reduced their working hours; 42.8% of Austrian, 41.5% of Swiss, and 17.3% of German participants closed their offices temporarily; 52.2% of respondents provided emergency service including pain management, restorations/temporaries, and denture repairs. A lack of access to FFP2/FFP3 (filtering facepiece) respirators was indicated by 59.4% Austrian, 38.0% German, and 11.7% Swiss dentists (*p* < 0.001). FFP2/FFP3 respirators were, when available, most frequently used in Austria (86.9%), followed by Switzerland (61.2%) and Germany (56.7%) (*p* < 0.001). Financial consequences could not be conclusively quantified by 58.6% of the participants. Most respondents in all partaking countries made use of governmental support. A lack of blueprints/guidelines resulted in heterogeneous working conditions. In consideration of a potentially high risk of infection in the dental setting, non-emergency dental treatments were largely suspended in all participating countries.

## 1. Introduction

The spreading of the new severe acute respiratory syndrome coronavirus-2 (SARS-CoV-2) across the world has led to a public health emergency of international concern and was declared a pandemic by the World Health Organization on 11 March 2020 [1]. The rapid increase of infections endangered to compromise intensive care capacities, which led to the implementation of mass quarantine measures of yet unknown extent all over the world. The first peak of SARS-CoV-2 infections in the German-speaking area of Europe was noted in March 2020. Therefore, the Austrian and Swiss governments implemented a strict lockdown on 16 March 2020. Germany followed on 22 March 2020 [2,3,4].

The human-to-human transmission of SARS-CoV-2 mainly occurs by droplets or direct contact of infectious material with the oral, nasal or ocular mucous membranes [5,6]. Most droplets triggered by speaking or coughing reach the ground within 1.8 m. As a consequence, the risk of infection can be greatly reduced by keeping a distance of >1 m [7]. First symptoms of the coronavirus disease 2019 (COVID-19) appear on average 5 days after exposure; 97.5% of patients develop symptoms within 11.5 days [8]. The transmission through contact with asymptomatic patients makes dissemination control of the virus challenging [6].

The dental setting poses several threats of infection. As SARS-CoV-2 is detectable in body fluids, direct contact with saliva/blood or indirect contact with contaminated surfaces or instruments represent a major source of infection [9,10]. In addition, airborne infections are to be considered in a dental setting in particular, since various dental instruments like ultrasonic scalers, 3-way syringes and high-speed handpieces generate aerosols. Viable SARS-CoV-2 could be detected in aerosols throughout 3 h [11]. Thirdly, dental treatments require a close proximity to the patient’s oral cavity, which puts the dental staff at risk for infection [12]. In spring 2020, these particular circumstances led to several recommendations or regulations for dental practitioners to reduce the risk of infection, which are partially still practiced. Non-urgent appointments had to be rescheduled. In situations where a postponement of the treatment would have resulted in a disadvantage for the patient, pre-treatment preventive measures were recommended. Patients had to be informed of the protective protocol on the phone, electronically, or in written form in advance. Masks and hygienic hand disinfection were obligatory. Screening for COVID-19 related symptoms in patients and staff via anamnesis and temperature measurement helped to separate suspected COVID-19 patients to prevent in-office spreading of the virus [13]. The number of patients simultaneously present in the office was reduced as far as possible, and sufficient distance had to be ensured, for example a one meter distance between seats in the waiting area. Magazines and demonstration models in the waiting room were removed as their proper disinfection could not be guaranteed [6,14,15].

Appropriate personal protective equipment for the dental staff including a mask, eyewear, gloves, a face shield, a disposable overgown, and hood, was highly recommended. Filtering facepiece respirators in FFP2 (94% minimal total filtration efficiency, corresponding to US N95 standard) or FFP3 (99% minimal total filtration efficiency, corresponding to US N99 standard) standards were recommended in case of contact with suspected or confirmed COVID-19 patients while performing aerosol-generating procedures [16,17]. To prevent infections while changing gloves, a double-glove technique with one long sleeved glove underneath was advised [14].

Further recommendations were pre-operative mouth rinses, reduction of aerosol producing actions, and the use of rubber dams. Mouth rinses with for example 1% hydrogen peroxide, 0.2% povidone iodine or 0.2% chlorhexidine are able to reduce aerosolized microbes. However, further studies are required with regard to their effectiveness against SARS-CoV-2 [14,18]. Reduction of aerosol-producing actions like ultrasonic scaling, restorative procedures or bracket/attachment removal with high speed handpieces decreases the risk of air-borne transmission [14]. If aerosol generating procedures cannot be avoided, high-volume salivary ejectors are necessary [15]. The use of rubber dams reduces aerosolized particles by 70% [19]. In order to reduce potentially infectious salivation, extraoral radiography should be preferred. [6]

Important post-treatment measures are hand washing with soap and/or disinfection with ethanol >60% to disrupt the external lipid layer of the virus [12]. Management of medical waste should be performed according to local regulations. All surfaces in the clinical area must be decontaminated according to a strict disinfecting protocol, and public facilities and community areas have to be disinfected on a regular basis with a special focus on door handles, chairs etc. [20]. Sufficient ventilation of the treatment rooms has to be ensured. In addition, an installation of a high efficiency particulate air (HEPA) filter may be considered [14].

In the first phase of the pandemic in particular, these recommendations were not accessible, and blueprints were missing to provide clear guidance for dentists. In addition, the lack of personal protective equipment complicated the working environment for dentists. The aim of this study was to investigate the working conditions of dentists in the German-speaking Central European regions during the first COVID-19 pandemic lockdown regarding protective protocols, working hours, and economic impact.

## 2. Materials and Methods

### 2.1. Design

A web-based survey consisting of 24 questions was designed using Research Electronic Data Capture (REDCap) (see File S1). The questionnaire contained questions regarding dentists’ field of work, working hours, treatments performed, personal protective equipment and protocols, and economic consequences and was distributed via a hyperlink by email. The addressees of the survey comprised dentists in Austria, Germany, Switzerland and South Tyrol. The email addresses were taken from the homepages of the local regulatory bodies sorted according to zip codes with the aim to reach the dental professionals as completely as possible. Regarding South Tyrol, the dental association of South Tyrol distributed the invitation to the survey via email to its members. One week after the first invitation, a reminder was sent to all addressees. A data protection statement was to be completed prior to the questionnaire. Only after the declaration of consent was given could the survey be answered. The survey period started on 12 August 2020 and ended on 5 November 2020.

### 2.2. Statistical Analysis

Continuous data is represented as mean ± standard deviation, and categorical data as absolute and relative frequencies. For group comparisons, the Chi-Square Test was used. *p*-values < 0.05 were considered statistically significant. All statistical calculations were performed using SPSS 26 (IBM Corp. Released 2019. IBM SPSS Statistics for Windows, Version 26.0. Armonk, NY, USA).

## 3. Results

### 3.1. Baseline Characteristics

For Austria, 2613 emails were sent out of which 129 could not be delivered; 15,804 German dentists received the invitation to the survey, while 1502 addresses were no longer up to date; 1446 emails to Swiss dental professionals could be delivered and 209 invitations failed. In total, 19,950 invitations were successfully delivered to Austrian, German, and Swiss dentists. The online survey was accessed 1925 times. In 63 out of 1925 cases (3.3%), access to the questionnaire was canceled before the declaration of consent was given. Out of the remaining 1862 cases (7%), 131 participants declined to accept the declaration of consent; 1731 dentists agreed to the data protection declaration and were included for further analysis. The mean age of the participants was 52.3 ± 15.6 years.

It was stated by 527/1.731 participants (30.4%) that they mainly worked in Austria. 60.8% corresponding to 1.053 participants mostly worked in Germany, and 6% (103/1731) in Switzerland. In 36 cases (2.1%), South Tyrol was specified as the predominant workplace. Twelve participants (0.7%) did not provide information on their place of work. Due to the low number of participants working in South Tyrol, a country-specific analysis of the situation in South Tyrol was not possible.

**Figure 1:**
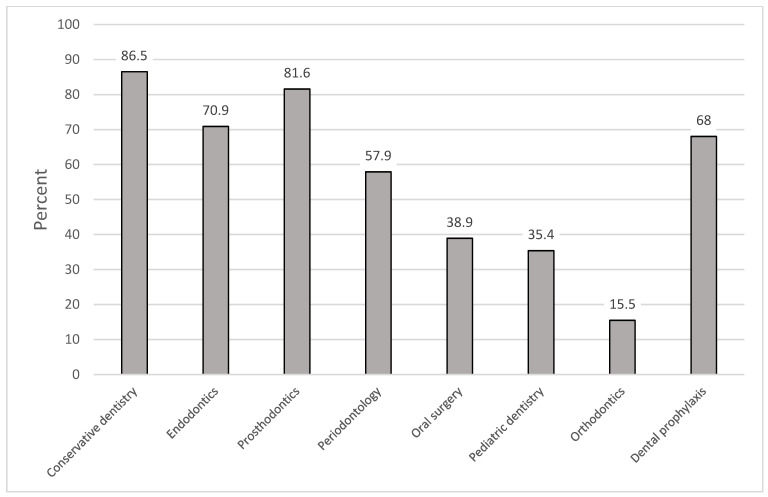
Distribution of field of work of participating dentists (multiple answers possible)**.**

More than 80% of participating dentists worked in the fields of conservative dentistry and in prosthodontics, followed by endodontics (70.9%) and periodontology (57.9%) (Figure 1). Less than 40% performed oral surgery, pediatric dentistry, or orthodontics (15.5%). Nearly 70% offered dental prophylaxis.

The majority of participants (82.2%) worked as self-employed in their own dental practice. 13.6% reported to work self-employed in a joint dental practice. 3.2% were in an employment relationship at a joint dental practice or an insurance institution, 2.8% at a university hospital, and 0.6% at a general hospital. 0.3% of participants were working exclusively as locum dentists, and 0.2% did not answer this question (multiple answers possible).

### 3.2. Working Hours during the First Pandemic Lockdown

The results on working hours showed that 10.6% of participating Austrian, 29.7% of participating German, and 3.9% of participating Swiss dentists did not report any change in their office hours during the first peak of the COVID-19 pandemic (*p* < 0.001). 53.7% of German, 45.5% of Austrian, and 11.7% of Swiss respondents reduced their working hours due to the COVID-19 pandemic.

The survey participants were asked whether and how long they decided to close their office temporarily; 82.7% of survey participants from Germany indicated that they had not interrupted their work during the first peak of the COVID-19 pandemic; 12.1% of German participants reported a closure of their offices during a period of 1 day to 3 weeks, 3.5% from 4 to 6 weeks, and 1.7% over 6 weeks. Concerning Austrian respondents, 57.2% did not report any practice closure, 22.6% interrupted their work in a timeframe of 1 day to 3 weeks, 15.2% from 4 to 6 weeks, and 5.1% for more than 6 weeks. Throughout the first lockdown, 58.5% of Swiss participating dentists continued to work, 29.8% closed their office for 4 to 6 weeks, and 11.7% interrupted their work for more than 6 weeks.

### 3.3. Treatments Performed during the First Peak of the Coronavirus Disease 2019 (COVID-19) Pandemic

The majority of participating dentists (52.2%) stated that extended emergency service including pain management, replacement of broken fillings or provision of temporaries, and denture repairs was provided; 56.3% of Swiss, 31.9% of Austrian, and 12.8% of German dentists performed solely pain management at some point of the COVID-19 pandemic (Table 1). Only 11.5% of all participants did not change their treatment modalities including dental prophylaxis. When analyzing this group by country of work, 17.3% of participants operating mainly in Germany, 2.8% of Austrian dentists, and 1.0% of the participants working in Switzerland performed all treatments without limitations (Table 1).

### 3.4. Personal Protective Equipment

Regarding personal protective equipment used before the COVID-19 pandemic (multiple answers possible), 90.4% of participants were using surgical masks as standard. 9.1% were using respirators in FFP2 or FFP3 standard beforehand.

**Figure 2:**
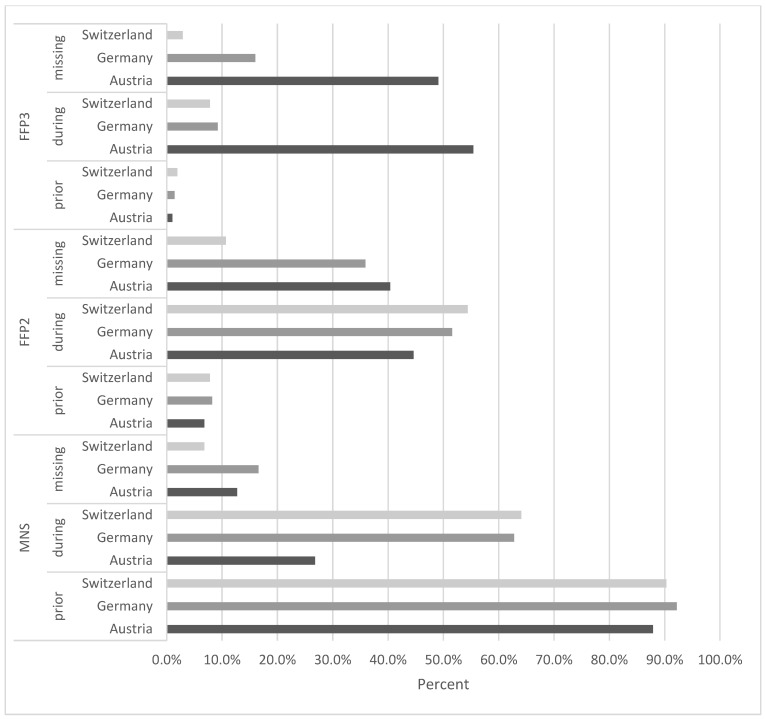
The standard use of protective masks (MNS = surgical masks, FFP2 (filtering facepiece) masks, FFP3 masks) prior to the COVID-19 pandemic, during the first peak of infections, and missing protective masks at the first peak of the COVID-19 pandemic.

At the first peak of the pandemic, the rate of dentists routinely using surgical masks dropped to 51.0% overall. Respirators in the FFP2 or FFP3 standard were most frequently used in Austria (86.9%), followed by Switzerland (61.2%) and Germany (56.7%) (*p* < 0.001). Regarding the use of FFP3 masks, clear statistical differences were shown (*p* < 0.001), as 55.4% of Austrian, 9.2% of German, and 7.8% of Swiss dentists were using FFP3 respirators as standard during the first lockdown (Figure 2).

After the occurrence of the first SARS-CoV-2 infections, 62.2% of Austrian, 43.7% of German, and 12.6% of Swiss study participants reportedly did not always have sufficient access to protective equipment (*p* < 0.001). The lack of access to respirators in the FFP2 and FFP3 standard during the first lockdown was reported by 59.4% Austrian, 38.0% of German, and 11.7% of Swiss respondents (*p* < 0.001) (Figure 2).

Prior to the COVID-19 pandemic, 96.8% of responding dentists were reportedly working with gloves and 1.7% were using a double-glove technique. At the first peak of the pandemic, using a second pair of gloves became more widespread with 6.8% in total (Austria 9.1%, Germany 5.2%, Switzerland 1.9%; *p* = 0.002).

Protective glasses were most commonly used in Switzerland (82.5% before and 88.3% at the peak of the pandemic), followed by Germany (74.3% and 76.1%) and Austria (64.3% and 66.0%, respectively). A more notable increase during the pandemic lockdown could be shown regarding the use of face shields and protective clothing. The use of face shields increased from 24.7% (prior to the pandemic) to 64.5% (during the pandemic) in Austrian, from 16.8% to 64.1% in German, and from 8.7% to 35.9% in Swiss respondents. The application of overgowns rose in Austria from 10.1% to 45.0%, in Germany from 8.8% to 23.8%, and in Switzerland from 7.8% to 16.5%. Protective hoods were utilized in 9.2% of the cases prior to the pandemic compared to 34.3% at the first lockdown (Austria 52.4%, Germany 25.7%, Switzerland 16.5%).

### 3.5. Protective Protocols during the COVID-19 Pandemic

In relation to protective measures integrated into the daily routine at the peak of the COVID-19 pandemic, the following measures were cross-nationally widespread: Reduced patients in the waiting area, periodic ventilation of the treatment rooms, specific information before or while entering the office, and disinfectants for the patients. These interventions were largely continued after the peak to the time of the survey. The reduction of aerosol-producing procedures and limitation of patient appointments were largely followed during the first pandemic lockdown, but were not continued to this extent afterwards. Other protective measures were integrated into the daily working routine to a lesser extent; 36.9% of Swiss, 13.6% of German, and 7.8% of Austrian participants used a rubber dam for aerosol-producing preparations with high-speed handpieces during the first lockdown (*p* < 0.001); 70.9% of Swiss, 55.6% of Austrian, and 55.7% of German participants screened their patients for COVID-19 related symptoms in form of a questionnaire (*p* = 0.011). Mandatory masks in the waiting area were pursued in 94.3% of cases in Austria, 84.0% in Germany, and 35.0% in Switzerland (*p* < 0.001) (Table 2).

### 3.6. Treated Patients

Up to the time of the survey, 6.9% of respondents treated patients with SARS-CoV-2 infection confirmed at the time of treatment. In a country comparison, the percentage of German dentists who treated patients with a diagnosed SARS-CoV-2 infection was highest with 7.3%, followed by Austrian participants with 5.9% and Swiss respondents with 4.9% (*p* = 0.022).

Of all partaking dentists, 922 were able to assess whether they treated infectious patients, and which tested positive at some point after the treatment; 9.9% of these participants (5.4% of total participants) treated infectious patients, who had not yet been diagnosed at the time of treatment. In a comparison by country of employment, there was no significant difference detectable (*p* = 0.629)

### 3.7. Economics

**Figure 3:**
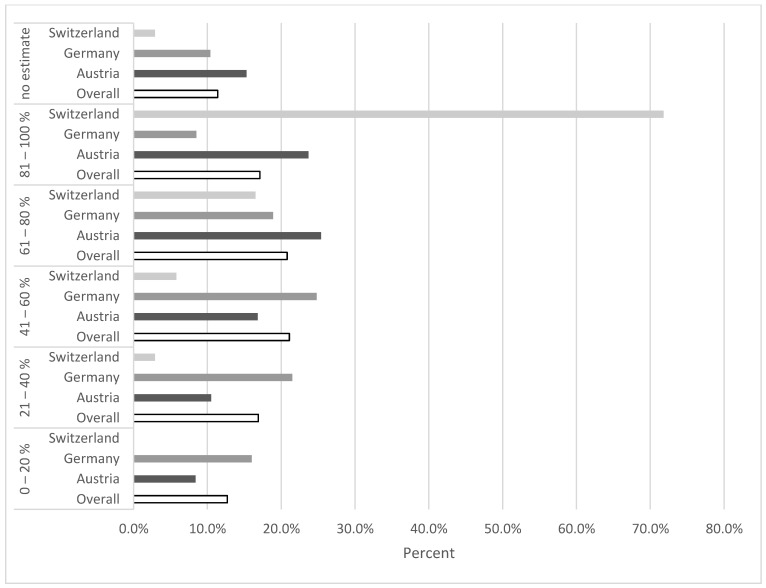
Estimated financial loss in the most unprofitable month during the first peak of the pandemic by partaking countries.

The COVID-19 pandemic and the countermeasures that the federal governments had enacted have resulted in substantial financial losses for most professions. The estimated financial loss in the most unprofitable month was rated in this study. 71.8% of Swiss participants estimated, that in the month associated with the greatest loss, their income dropped by 80–100%; 23.4% of Austrian respondents and 8.5% of German dentists assessed their financial loss as equally drastic (Figure 3).

Consequences resulting from the financial losses could not be conclusively quantified by the majority of participants (58.6%) up to the point of the survey; 36.4% of German, 39.4% of Austrian, and 51.5% of Swiss dentists estimated that operations could be resumed without consequences. The loss of employees was stated in 6.5% of cases, the dismissal of staff was required in 5.9% of cases, 8.5% of participants found long-term restricted office hours to be necessary, and 11.7% of respondents were going to establish surcharges for patients.

**Figure 4:**
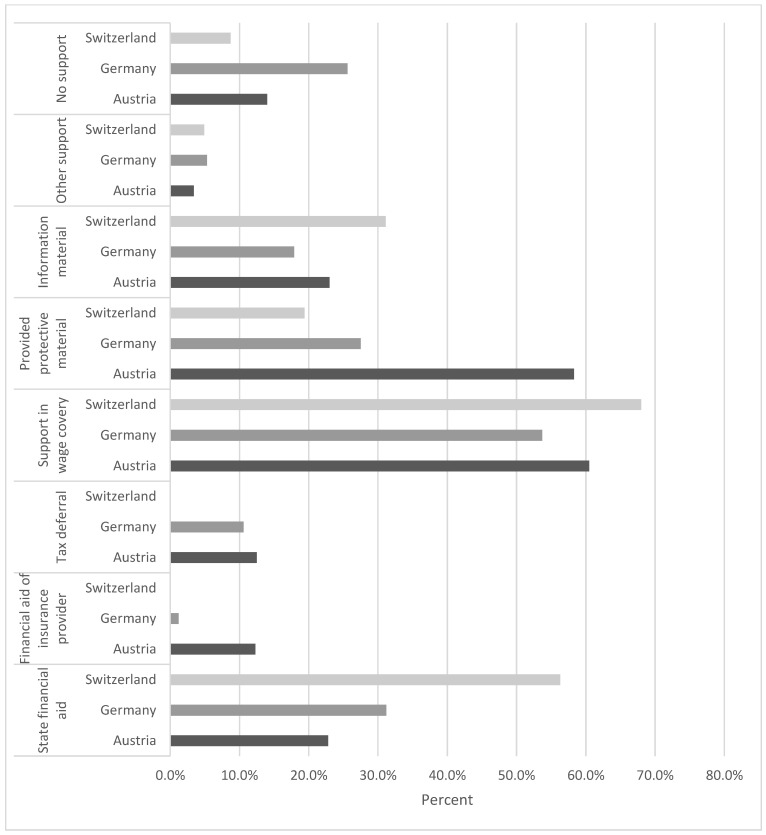
Support options used by dentists in Austria, Germany and Switzerland during COVID-19 pandemic.

Support options in various forms were obtained by the majority of participants during the first peak of SARS-CoV-2 infections; 21.1% stated that they did not receive any aid. A measure that was used by a great part of respondents in all partaking countries was financial aid to cover wage costs; 60.5% of Austrian, 53.7% of German, and 68% of Swiss survey participants were supported accordingly (Figure 4).

Finally, the participants were asked about their satisfaction with the support they received from their professional association; 55.88% (±26.77) of Swiss, 39.19% (±28.45) of Austrian, and 37.80% (±27.35) of German participants felt being adequately represented by their regulatory bodies.

## 4. Discussion

The COVID-19 crisis is a great challenge in various respects. Dentists had to find a way to guarantee basic care for patients and, at the same time, to prevent in-office infections. State-imposed recommendation and/or regulations were initially not available, changed quickly, and differed from one country to another. Therefore, our presumption of a lack of protective protocols or therapeutic concepts was confirmed by significant cross-national differences in all performed treatments and for the most protective measures integrated into the daily routine in the dental setting. This may be a result of different governmental recommendations. As Switzerland banned all medical treatments that were not absolutely necessary on 16 March 2020 [21], the number of Swiss dentists who provided pain emergency service only at some time during the COVID-19 pandemic was significantly higher when compared to Austria and Germany. On 15 March 2020, the Austrian Dental Association recommended that dentists only provide emergency treatments which could not be rescheduled to a later point [22]. Recommendations issued by the German dental association stated that dentists should decide together with their patients whether a planned treatment was really urgent under the prevailing circumstances or whether it could be postponed for the time being with particular caution regarding vulnerable population groups [23]; 17.3% of German participants performed all treatments without limitation even at the peak of the first wave of infection, compared to 2.8% of Austrian respondents and 1.0% of Swiss dentists. A study concerning awareness, protective measures, and economic effects of dentists in Switzerland and Liechtenstein came to similar results. Wolf et al. described that less than 2% of participants reported no change in their work processes. 49.66% continued working with additional hygiene and protective measures [24].

Although preventive lockdowns of dental offices were called for, all participating countries limited government-imposed closures of dental offices to cases of infection on the grounds that dental treatments represent an essential element of health care. Nevertheless, 42.8% of Austrian, 41.5% of Swiss, and 17.3% of German survey participants closed their offices temporarily. A survey conducted among Polish dentists showed that 71.2% of participating dentists suspended their work in the first peak of the COVID-19 pandemic, which the authors attribute to a significant decrease in the number of patients and insufficient access to personal protective equipment, as described by 75.3% of respondents [25]. A reduction of the number of patients and a lack of adequate personal protective measures could be shown in this study as well.

Significant differences could also be shown regarding the utilization of personal protective equipment. In Austria, respirators in FFP2 or FFP3 standard were used by 86.9% of participants compared to 56.7% of German and 61.2% of Swiss participants as standard at the first peak of infection (*p* < 0.001). Respirators in FFP3 standard were prioritized in Austria only (Figure 2). The difference in the utilization of masks could not be attributed to the indifferent access, since the lack of respirators was described mostly by Austrian participants, followed by German and Swiss respondents. In most COVID-19 related surveys dentists were asked about using FFP2/3 masks resulting in heterogenous outcomes. In North Italy, an area that suffered from high numbers of infection and the impending overload of the health care system, 58.84% of partaking dentists used respirators after the occurrence of the first infections; however, access to FFP2/3 masks was not evaluated in this study [26]. A survey conducted in dental staff in Norway showed that 22.9% of participants used a respirator in FFP2/3 standard while treating patients without suspected infection [27]. One aspect of this variation could be the different sensitization in regard to the COVID-19 pandemic in each country. Countries with a high incidence early in the pandemic, like Italy, or countries that were implicated in the dissemination of the virus, like Austria, possibly had a higher awareness during the first phase of the pandemic.

Regarding the treatment of patients suffering from COVID-19, there is a consensus with regard to specific recommendations. Infectious patients should be treated in treatment centers at clinics or at specialized practices. In this regard, the incubation period and the possibly asymptomatic course of the disease pose a problem; 5.4% of all participants (with no significant difference in cross-country comparison) stated that they had treated patients who had a current infection with SARS-CoV-2 at the time spent in the office but were diagnosed only after the treatment. This demonstrates the importance of protective measures to prevent in-office infections.

Although Swiss dentists estimated their financial loss most drastically (Figure 3), more than half of the respondents from Switzerland believed that procedures could be resumed without consequences after the pandemic; 36.4% of German and 39.4% of Austrian respondents shared this opinion. In all participating countries the most frequently used support option was support in wage coverage; 55.88% (±26.77) of Swiss, 39.19% (±28.45) of Austrian, and 37.80% (±27.35) of German participants felt adequately represented by their regulatory bodies. These figures suggest that the dentist would have appreciated more support from their regulatory bodies and might represent a thought-provoking impulse for professional representatives. Up to the time of the survey, 58.6% of participants were not able to conclusively assess their financial losses and the consequences following the first peak of the pandemic. These uncertainties regarding their occupational future may stem from the fact of the emerging second wave of infection at the time of the survey. In addition, for panel dentists a concluding calculation is only possible after the end of the quarter.

There are several limitations to this online survey. Although we have considered all available sources to retrieve the maximum email addresses, our final database containing 19,950 valid email addresses is certainly not complete. Furthermore, there are—presumably rather few—colleagues who may not have an email account at all, which automatically excluded them from being contacted. No efforts were made to contact dentists by conventional mail in this online survey, which may result in an overrepresentation of technophilic dentists. Another limitation is the representativeness of the study group; 527 Austrian participants compared to ~5000 Austrian dentists represent the basic population better than 1053 German participants compared to ~70,000 German dentists or 103 Swiss participants compared to ~7000 Swiss dentists. A higher number of participants would increase the significance of the results.

In conclusion, it became apparent that there were no uniform blueprints and guidelines to be followed, which resulted in heterogeneous working conditions of dentists during the first peak of the COVID-19 pandemic. Although the high risk of infection in the dental setting was acknowledged, dental treatments as main source of infection were largely suspended in all participating countries. According to a survey conducted by the Austrian dental association addressing mainly self-employed dentists in Austria, 2% of employees and 1% of dentists were infected with SARS-CoV-2 during the first peak of infections [28]. It was not evaluated if the infections occurred within the working environment. The low number of infections among Austrian dentists during the first peak of infection indicates that effective protective measures, as for example a particularly high percentage of dentists using respirators in the FFP2/3 standard were able to compensate for the increased risk of infection. The number of infections in dental staff were evaluated in other European countries as well, which will allow for conclusions on the efficiency of the protective measures that came into effect.

## Figures and Tables

**Table 1:** Comparison of performed dental treatments during the first coronavirus disease 2019 (COVID-19) pandemic lockdown in Austria, Germany and Switzerland, multiple answers possible.

Treatment	Austria		Germany		Switzerland		
	n	%	n	%	n	%	*p*
Telemedical care	88	16.7%	27	2.6%	13	12.6%	<0.001
Pain management exclusively	168	31.9%	135	12.8%	58	56.3%	<0.001
Extended emergency service	329	62.4%	503	47.8%	58	56.3%	<0.001
All treatments except professional dental hygiene	94	17.8%	371	35.2%	2	1.9%	<0.001
All treatments without limitations	15	2.8%	182	17.3%	1	1.0%	<0.001

**Table 2:** Protective measures integrated into daily routine during the first COVID-19 lockdown in Austria, Germany, and Switzerland.

Protective Measure	Austria		Germany		Switzerland		
	n	%	n	%	n	%	*p*
Reduced capacity in waiting area	493	93.5%	989	93.9%	95	92.2%	0.785
Periodic ventilation	488	92.6%	981	93.2%	101	98.1%	0.124
Information at entrance	497	94.3%	1005	95.4%	92	89.3%	0.026
Disinfection for patients	495	93.9%	964	91.5%	101	98.1%	0.022
Reduction of aerosols	449	85.2%	808	76.7%	81	78.6%	<0.001
Limitation of appointments	438	83.1%	740	70.3%	78	75.7%	<0.001
Preparation with rubber dam	41	7.8%	143	13.6%	38	36.9%	<0.001
Patient screening with questionnaire	293	55.6%	587	55.7%	73	70.9%	0.011
Mandatory masks in the waiting area	497	94.4%	884	84.0%	36	35.0%	<0.001
Body temperature measurement in patients	170	32.3%	100	9.5%	45	43.7%	<0.001
Body temperature measurement in staff	135	25.6%	100	9.5%	29	28.2%	<0.001
Installation of HEPA-Filter	42	8%	69	6.6%	16	15%	0.004

## Data Availability

All data generated in this study is contained within this publication.

## Supplementary Materials

The following are available online at https://www.mdpi.com/2227-9032/9/3/364/s1, File S1: Survey.
