# Supplementary material for: Dentists’ Working Conditions during the First COVID-19 Pandemic Lockdown: An Online Survey
Source: Healthcare (Basel). 2021 Mar 23;9(3):364. doi: 10.3390/healthcare9030364 (PMC8004942; doi:10.3390/healthcare9030364)
Supplement: Supplementary file 1 [file healthcare-09-00364-s001.pdf]

Questionnaire study on working conditions of dentists during the first COVID-19 lockdown

1.) In which country do you predominantly work in?

a. Austria

b. Germany

c. Switzerland

d. South-Tirol

2.) How old are you? (Drop-down 23 - 85 years)

3.) In which field of work do you mainly work in (multiple answers possible)?

a. Conservative dentistry

b. Endodontics

c. Prosthodontics

d. Periodontology

e. Oral surgery

f. Pediatric dentistry

g. Orthodontics

h. Dental prophylaxis

4.) What employment relationship are you in (multiple answers possible)?

a. Self-employed in your own practice

b. Self-employed in a community practice

c. Employment at a joint practice (e.g. further training assistant), outpatient clinic (e.g. an insurance company), or the like.

d. Employment at a university clinic

e. Employment at a general hospital

f. Exclusively acting as substitute dentist (in A: Wohnsitz-Zahnarzt)

g. no information

5.) How had your workflow changed due to the COVID-19 pandemic (during the lockdown) with regard to your **working hours** (multiple answers possible)?

a. No change in working hours
b. Extended office hours
c. Restricted office hours
d. Emergency service only in your own practice
e. Participation in central emergency services
f. Temporary practice closure
g. Employment relationship with unchanged weekly hours
h. Employment relationship with reduced weekly hours
i. Employment relationship with increased weekly hours
j. No information

6.) If you closed your practice completely during the lockdown, how long did it last?

a. Drop-Down (1 - 10 weeks; no practice closure, practice is still being closed)

7.) How had your workflow changed due to the COVID-19 pandemic (during the lockdown) with regard to the type of treatments carried out (multiple answers possible)?

a. No treatment due to interruption of work
b. Telemedical care
c. Pain management exclusively (e.g. extractions, trepanations, incisions, etc.)
d. Extended emergency service (including pain management, replacement of broken fillings, provision of temporaries, denture repairs)
e. All treatments as before the pandemic except professional dental hygiene
f. All treatments as before the pandemic without restrictions
g. no information

8.) With regard to personal protective equipment: Which protective equipment did you use as standard **BEFORE** the COVID-19 pandemic for every patient (multiple answers possible)?

a. Surgical mask
b. FFP1 mask
c. FFP2 mask

d. FFP3 mask

e. Eye protection

f. Face shield

g. Gloves (one layer)

h. Gloves (double layer)

i. Protective clothing

j. Protective hood

k. Other protective equipment

9.) Which personal protective equipment did you use for every patient **AT THE PEAK** of the COVID-19 pandemic (multiple answers possible)?

a. Surgical mask

b. FFP1 mask

c. FFP2 mask

d. FFP3 mask

e. Eye protection

f. Face shield

g. Gloves (one layer)

h. Gloves (double layer)

i. Protective clothing

j. Protective hood

k. Other protective equipment

10.) Which protective measures did you integrate into your daily routine **AT THE PEAK** of the COVID-19 pandemic (multiple answers possible)?

a. Information at the entrance

b. Screening questionnaire in which, for example, symptoms, contact with infected people, stay at risk area, etc. are queried

c. Telephone and / or online consultations before the appointment

d. Measurement of the patient's body temperature

e. Measurement of the body temperature of the employees

f. Disinfectants for the patients

g. Mandatory masks in the waiting area

h. Limitation of appointments

i. Reduced capacity in waiting area

j. Reduction of aerosol forming activities (e.g. oral hygiene with ultrasound)

k. Periodic ventilation of the practice rooms

l. Installation of ventilation systems

m. Using rubber dam for aerosol-producing preparations

n. None of the measures mentioned

11.) Which protective measures have you **CURRENTLY** integrated into your daily routine (multiple answers possible)?

a. Information at the entrance

b. Screening questionnaire in which, for example, symptoms, contact with infected people, stay at risk area, etc. are queried

c. Telephone and / or online consultations before the appointment

d. Measurement of the patient's body temperature

e. Measurement of the body temperature of the employees

f. Disinfectants for the patients

g. Mandatory masks in the waiting area

h. Limitation of appointments

i. Reduced capacity in waiting area

j. Reduction of aerosol forming activities (e.g. oral hygiene with ultrasound)

k. Periodic ventilation of the practice rooms

l. Installation of ventilation systems

m. Using rubber dam for aerosol-producing preparations

n. None of the measures mentioned

12.) Have you treated patients with a confirmed COVID-19 infection?

a. Yes

b. No

c. No information

13.) Have you treated infectious patients, which were tested positive at some point after the treatment?

a. Yes

b. No

c. Currently no cases known

d. No information

14.) Have you treated patients with suspected COVID-19 infection (symptoms such as increased body temperature, cough, etc. or patients under quarantine)?

a. Yes

b. No

c. No information

15.) Please estimate your financial loss in the most unprofitable month (how many percent did you earn less compared to the situation before the COVID-19 crisis)?

a. 0-20%

b. 20-40%

c. 40-60%

d. 60-80%

e. 80-100%

f. Cannot be estimated or finally calculated yet

16.) What are the consequences that resulted of these financial losses (multiple answers possible)?

a. I didn't suffer any financial losses

b. No consequences, operations can be resumed as usual after the COVID-19 crisis

c. Employees have left

d. Termination of employees was required

e. Long-term restricted office hours

f. Fee increase / surcharges for patients

g. Not yet conclusively assessable

h. Other consequences

17.) Which support options did you obtain during der COVID-19 pandemic (multiple answers possible)?

a. Financial support from the state

b. Financial support from an insurance provider

c. Tax deferral

d. Financial support to cover the wage costs of the employees ("Kurzarbeit")

e. Provided protective material

f. guidelines / information material

g. I obtained other support

h. I did not receive any support

.

18.) Have you always had sufficient access to personal protective equipment since the first COVID-19 infections?

a. Yes

b. No

c. No information

19.) If no, which protective equipment was/were missing?

a. Surgical mask

b. FFP1 mask

c. FFP2 mask

d. FFP3 mask

e. Eye protection

f. Face shield

g. Gloves (one layer)

h. Gloves (double layer)

i. Protective clothing

j. Protective hood

k. Other protective equipment

20.) Do you currently have sufficient access to personal protective equipment (again)?

a. Yes

b. No

c. No information

21.) If no, which personal protective equipment is/are you currently missing?

a. Surgical mask

b. FFP1 mask

c. FFP2 mask

d. FFP3 mask

e. Eye protection

f. Face shield

g. Gloves (one layer)

h. Gloves (double layer)

i. Protective clothing

j. Protective hood

k. Other protective equipment

22.) Which personal protective equipment do you use for every patient **AT THE MOMENT** (multiple answers possible)?

a. Surgical mask

b. FFP1 mask

c. FFP2 mask

d. FFP3 mask

e. Eye protection

f. Face shield

g. Gloves (one layer)

h. Gloves (double layer)

i. Protective clothing

j. Protective hood

k. Other protective equipment

23.) With the declining infection numbers, some state-induced measures have now been gradually withdrawn. How is this affecting your working hours at this point in time?

a. Return to office hours as before the pandemic

b. Other ordination times, but the same total number of hours per week as before

c. Extended office hours, i.e. an increase in the total number of hours per week

d. Ongoing restricted office hours, i.e. reduction of the total number of hours per week

e. Persistent practice closure

f. Not specified

24.) Do you feel adequately represented by your professional association? (Scale 0-10, 0: not at all to 10: optimal)

a. Drop-Down (0 - 10, not specified)
